# Research on Angle-Adaptive Look-Ahead Compensation Method for Five-Degree-of-Freedom Additive Manufacturing Based on Sech Attenuation Curve

**DOI:** 10.3390/mi17040423

**Published:** 2026-03-30

**Authors:** Xingguo Han, Wenquan Li, Shizheng Chen, Xuan Liu, Lixiu Cui

**Affiliations:** 1 College of Mechanical and Control Engineering, Guilin University of Technology, Guilin 541006, China; hanxingguo2004@163.com; 2 Guangxi Key Laboratory of Special Engineering Equipment and Control, Guilin 541004, China; cuilixiu@guat.edu.cn; 3 University Engineering Research Center of Non-Standard Intelligent Equipment and Process Control Technology, Guilin 541004, China; 17863642183@163.com; 4 Guangxi Region Precious Metal Materials Advanced Process Research Center, Guilin 541004, China; 5 School of Mechanical Engineering, Guangxi University, Nanning 530004, China; 2411391011@st.gxu.edu.cn

**Keywords:** additive manufacturing, look-ahead compensation, sech function, 3d printing

## Abstract

To address over-extrusion and forming defects at path corners caused by path overlap in additive manufacturing, this paper proposes an angle-adaptive look-ahead compensation algorithm based on a Sech attenuation curve. This method establishes a mapping model between the path angle and the adaptive look-ahead distance of the overlapping area, aiming to eliminate the material accumulation at the corner by precisely identifying the overlapping area and modulating the flow rate. By building a Beckhoff five-axis 3D-printing device and relying on the TwinCAT control platform, the compensation triggering logic based on PLC real-time Euclidean distance calculation was realized, and a slicing software with dynamic bias compensation was also developed. Experiments were conducted on triangular samples with extreme acute angles of 5°, universal acute angles of 85°, and 90° straight angles for printing verification. The results show that this algorithm can effectively suppress the material over-extrusion and accumulation at the path overlap in multiple angles, achieving a smooth transition of the sharp corners in the printed contour. The research confirms that the algorithm proposed in this study, together with the integrated software and hardware system, can ensure the forming accuracy of extreme and conventional geometric features while also guaranteeing the printing efficiency, providing an important reference for ensuring the quality coordination control of the formation process of extreme geometric features in additive manufacturing.

## 1. Introduction

Additive manufacturing’s low costs and material versatility have driven its adoption across aerospace, medical, and automotive sectors [1]. This technology melts and extrudes thermoplastic filaments such as polylactic acid (PLA) and acrylonitrile butadiene styrene (ABS) through a heating nozzle and builds up the structure layer by layer along a predetermined path, featuring low equipment costs, wide material selection, and simple operation [2,3]. In the actual manufacturing process, FDM technology can achieve the integrated formation of complex internal geometric structures, providing great convenience for the production of lightweight designs and topological optimization components [4]. However, the manufacturing accuracy and quality consistency of FDM parts remain the main bottleneck restricting the transition to industrial-grade functional components, especially in the precise forming of complex paths, where microscopic defects in the process often lead to significant performance losses [5].

Among various manufacturing defects, the accumulation of material at the path corners is one of the key factors affecting the forming accuracy [6]. The causes of this problem can be analyzed from two perspectives: from the kinematic perspective, the nozzle needs to decelerate and turn at the corner, but the response of the extrusion system lags, resulting in a mismatch between material transportation and movement speed, causing local material overabundance [7]; and from the thermal physical perspective, the increase in the residence time at the corner will lead to local heat accumulation, which, in turn, causes the molten pool to overheat and deform [8]. This local material overabundance not only causes the geometric dimensions of the part to deviate from the design value [9], but also significantly increases the surface roughness and even reduces the tensile strength and fatigue life of the part due to the concentration of interlayer stress [10,11].

Regarding the phenomenon of corner material accumulation and the methods for improving surface quality, the existing research mainly focuses on three dimensions: path planning, process regulation, and real-time control. In terms of path optimization, researchers have proposed arc transition as an alternative to sharp corners [12] and speed optimization strategies [13], and often sacrifice speed to enhance corner quality [14,15]. At the process level, the coupling relationship between nozzle temperature, cooling rate, and extrusion ratio is optimized to improve surface formation quality [16,17]. In terms of control algorithms, proportional–integral–differential (PID) control [18], model predictive control [19], and nonlinear smoothing algorithms [20] are adopted to improve path sharpness issues. The existing look-ahead algorithms mainly focus on optimizing printing speed [21]; these methods usually improve forming quality at the expense of printing speed, and it is difficult to achieve universal smoothing effects under complex geometric angles.

From the perspective of rheology, molten polymers exhibit significant shear thinning and viscoelastic behaviors [22]. The pressure transmission during the extrusion process has typical nonlinear lag characteristics, namely “extrusion expansion” and a pressure relaxation effect [23]. The traditional linear compensation model cannot accurately describe this physical process that starts smoothly at the forward boundary of the turning angle and decays rapidly at the vertex. Therefore, the introduction of a nonlinear function with higher-order continuity for interpolation compensation becomes an inevitable choice.

To address the aforementioned issues, this paper proposes an angle-adaptive forward compensation method based on the Sech function. The research was conducted on a five-axis simultaneous-motion printing platform in a three-axis motion mode, focusing on the physical verification and geometric adaptability design of the algorithm. By establishing an algorithm for the correlation between the forward limit distance and the rotation angle, and by using the Sech function as the attenuation factor to construct the extrusion gradient, a smooth transition of extrusion volume from 1.0 to 0.1 at the rotation point was achieved, providing a new theoretical solution for high-precision FDM manufacturing.

## 2. Angle-Adaptive Look-Ahead Compensation Algorithm

### 2.1. Reason Analysis

In the Fused Deposition Modeling (FDM) process, the ideal printing path is regarded as a continuous filling body with a constant width and height. When the nozzle performs linear movement, the filament delivery rate of the extrusion system is in dynamic equilibrium with the feed rate of the nozzle. However, when the path undergoes a direction change (i.e., there is a turn), the geometric layer overlap becomes the fundamental cause of material accumulation.

#### 2.1.1. Geometric Overlap Analysis

**Figure 1:**
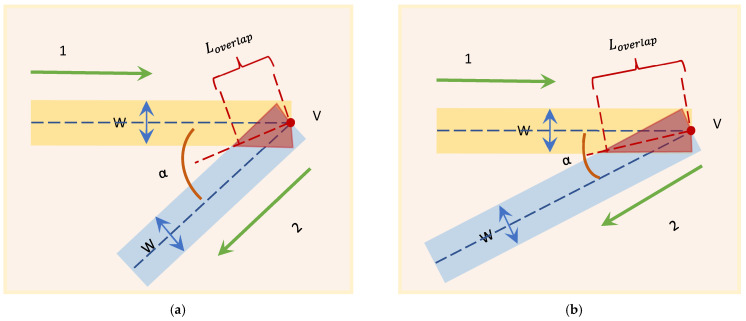
Comparison of geometric overlapping models at the path corners: (**a**) Overlapping area at *α* = 45°; and (**b**) overlapping area at *α* = 30°. The green arrow indicates the movement direction of paths 1 and 2; the yellow area represents the line width (W) of path 1, the blue area represents the line width (W) of path 2; the red shaded area represents the geometric overlapping area.

As shown in Figure 1, consider two printing paths of the same width that intersect at an angle (internal angle) at a vertex. Geometrically, near the vertex, the two paths will form a rhombus-shaped overlapping area (Overlap Region).

The length $L_{overlap}$ of the central line of this overlapping area is the distance from the vertex to the boundary of the overlapping zone, and it can be derived through trigonometric geometric relationships:

(1) $$L_{overlap}=\frac{W/2}{\sin\left(\alpha /2\right)}$$

From a physical perspective, when the nozzle moves along path 1 to the vertex V and then switches to path 2, if the extrusion pressure remains constant, the amount of material filled per unit area within the rhombic overlapping region will reach twice the theoretical value. As the angle decreases, $L_{overlap}$ increases nonlinearly, which means that the sharper the corner, the longer the over-extrusion area caused by the geometric overlap.

#### 2.1.2. Analysis of the Accumulation Effect

Apart from geometric overlap, the dynamic characteristics of the extrusion system further exacerbate the deterioration of quality at the turning points. Due to the significant viscoelasticity of the molten polymer, there is a time lag in the pressure response within the nozzle. At a constant feed rate, even if the nozzle does not decelerate at the turning point, the sudden change in the flow vector caused by the path turn will form a temporary high-pressure zone at the apex.

The “insufficient space margin” caused by geometric overlap and the “excessive material supply” resulting from pressure lag are coupled with each other, eventually forming visible protrusions at the corners. This stacking effect not only damages the dimensional accuracy of the parts but also causes physical interference between the nozzle of the next layer and the protrusion of the previous layer during printing, triggering vibrations and even printing failure. Therefore, establishing an advanced algorithm that can sense the change in angle α and adjust the pressure in advance is the core of solving this problem.

### 2.2. Algorithm Model

#### 2.2.1. Compensation Strategy

The above analysis reveals the physical essence of corner accumulation: the spatial constraints caused by geometric overlap, combined with the viscoelastic pressure lag, result in excessive deposition of the material at the corner vertices. To suppress this phenomenon, it is necessary to actively reduce the extrusion volume before the nozzle reaches the corner, leaving sufficient response time for pressure release and material contraction. This “pre-emptive perception, progressive adjustment” control strategy is called look-ahead compensation. Its core lies in predicting the future geometry of the path in advance and adjusting the process parameters in advance to avoid the lag of traditional feedback control.

However, the effectiveness of look-ahead compensation is highly dependent on the morphological design of the extrusion gradient curve. The traditional linear interpolation method uniformly reduces the extrusion amount from the normal rate to the corner relief rate. This constant slope decay pattern is fundamentally incompatible with the nonlinear rheological properties of the polymer melt. Specifically, the linear compensation is too abrupt at the entry end, and the sudden drop in extrusion amount leads to insufficient material supply in the look-ahead initial section, resulting in under-extrusion marks; while near the corner apex, the slope of the linear decay appears too gentle and is unable to completely release the accumulated pressure in the nozzle cavity within the limited look-ahead distance, resulting in residual accumulation at the apex. More critically, the fixed-slope linear model cannot adapt to the geometric overlap changes under different corner angles. Acute angles and obtuse angles use the same compensation intensity, inevitably causing insufficient compensation at acute angles and excessive compensation at obtuse angles.

Therefore, the ideal compensation curve should possess the following characteristics:

- The cutting end is gentle: During the initial stage of compensation extrusion, maintain a high extrusion rate and achieve a smooth transition.
- The end is steep: When approaching the geometric overlapping vertex, the extrusion rate needs to sharply decrease to counteract the double filling effect caused by geometric overlap and the delay in motor rotation.
- Angle adaptation: The compensation distance is dynamically adjusted according to the angle α.

#### 2.2.2. Angle-Adaptive Forward Compensation Algorithm

In order to adapt the algorithm to the path corner with different topological complexity, an extended look-ahead model based on the diagonal length $L_{overlap}$ of the angular bisector is established. The equation for the look-ahead limit distance $D_{la}$ is as follows:

(2) $$D_{la}=\left\{\begin{array}{c} \frac{W}{2\sin\left(\alpha /2\right)}+W,\;\alpha <45{}^{\circ}\\ \;2W,\;\alpha \geq 45{}^{\circ} \end{array}\right.$$

Among them, W represents the preset line width, and it is the angle between the two path centerlines.

The design of this model encompasses two core dimensions:

- Geometric compensation distance: By using sin(α/2), the farthest physical boundary of the overlapping area along the angle bisector direction is mapped out. Compared with the simple linear look-ahead, this term can ensure that the look-ahead range automatically extends with the expansion of the overlap area when turning at an acute angle.
- Response delay compensation: Considering the dynamic response delay of the extrusion system, the final look-ahead limit distance $D_{la}$ adds an additional compensation amount of line width W to the diagonal length. Additionally, for the α≥45° case, the algorithm adopts a fixed double-line-width compensation to ensure the robustness of the algorithm under different angle topologies.

### 2.3. Extrusion Control Based on the Sech Function

#### 2.3.1. Construction of the Sech Attenuation Factor Model

To achieve a seamless transition from the normal extrusion state to the micro-extrusion state with a sharp corner, this study proposes a smoothing control algorithm based on the Sech function. This algorithm uses the Sech function to construct a nonlinear flow attenuation factor, enabling the extrusion volume to be adaptively adjusted according to the position of the nozzle.

**Figure 2:**
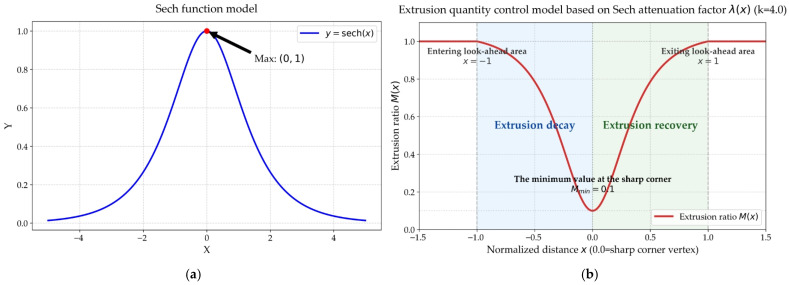
Construction of a traffic control model based on the Sech function: (**a**) The mathematical characteristics of the Sech function; (**b**) the extrusion volume control process based on the Sech attenuation factor.

The hyperbolic secant function $f(x)=\mathrm{sech}(x)=\frac{1}{cosh(x)}$ possesses excellent mathematical properties, as shown in Figure 2a: it attains its maximum value of 1 at x=0 and has a derivative of 0. As $\left|x\right|$ increases, it smoothly decays to 0. Utilizing this characteristic, we define the normalized flow attenuation factor λ(x):

(3) $$\lambda (x)=\frac{\mathrm{sech}(k\cdot x)-\mathrm{sech}(k)}{1-\mathrm{sech}(k)},\;x\in [0,1]$$

In the development of the nonlinear extrusion compensation strategy, the Sech function was selected in this study not simply because of its mathematical novelty, but because it provides a more suitable balance among compensation continuity, parameter simplicity, and real-time implementability in corner regions. Although many existing nonlinear compensation methods can also achieve a certain degree of extrusion adjustment, the five-degree-of-freedom fused deposition process investigated here imposes additional requirements on the compensation function. It must not only describe the variation trend of extrusion demand near corners, but also remain computationally lightweight enough for practical real-time execution in the PLC-based control framework. Compared with nonlinear compensation forms that rely on piecewise construction, local switching, or higher-order fitting, the Sech function is more appropriate for the present application scenario. Its mathematical expression is compact and involves fewer parameters, which makes it easier to integrate into the real-time control procedure while maintaining a continuous compensation output and avoiding extra control fluctuations caused by abrupt function transitions.

From the perspective of curve characteristics, the Sech function varies smoothly around its peak and decays continuously on both sides, without introducing obvious discontinuities. This feature is consistent with the practical behavior of material extrusion in fused deposition, where the extrusion response does not instantly follow rapid changes in motion speed. When the nozzle decelerates or changes direction near a corner, the melt flow state usually cannot adjust synchronously at the same rate, which makes local material accumulation more likely to occur. Under such conditions, a continuously varying compensation function is helpful for alleviating the over-extrusion caused by the mismatch between speed variation and extrusion response. The experimental validation at 5°, 85°, and 90° corners in this study also indicates that this functional form can meet the compensation requirements under different angular conditions.

It should also be noted that, in the implementation of the proposed algorithm, the scaling factor K was set as a fixed constant. The purpose was not to emphasize parameter tuning itself, but to first evaluate the basic applicability of the Sech curve to different corner conditions without introducing an additional parameter-optimization process. Under this setting, the compensation form remains unchanged and does not require reconstruction of the model for each geometric feature, which is advantageous for engineering implementation. At the same time, K determines the width of the compensation interval, which means that it still has potential for further optimization. Since different filament materials exhibit different viscoelastic and flow-lag characteristics, future studies may further calibrate K for specific materials, such as PLA and TPU, in order to obtain more precise extrusion matching. Therefore, the advantage of the Sech function in this work does not lie in claiming universal superiority over all nonlinear compensation methods, but rather in its ability to achieve a suitable balance among compensation continuity, implementation simplicity, and real-time executability for the five-degree-of-freedom corner-compensation scenario considered in this study.

#### 2.3.2. Dynamic Control of Extrusion Rate

Based on Equations (2) and (3), a control model for the extrusion ratio M(d) can be constructed. This model divides the extrusion behavior into a gradient control zone and a constant extrusion zone according to the real-time physical distance d between the nozzle and the sharp corner:

(4) $$M(d)=\left\{\begin{array}{c} M_{\max}-\left(M_{\max}-M_{\min}\right)\cdot \lambda \left(\frac{d}{D_{la}}\right),\;0\leq d\leq D_{la}\\ \;M_{\max},\;d>D_{la} \end{array}\right.$$

**Figure 3:**
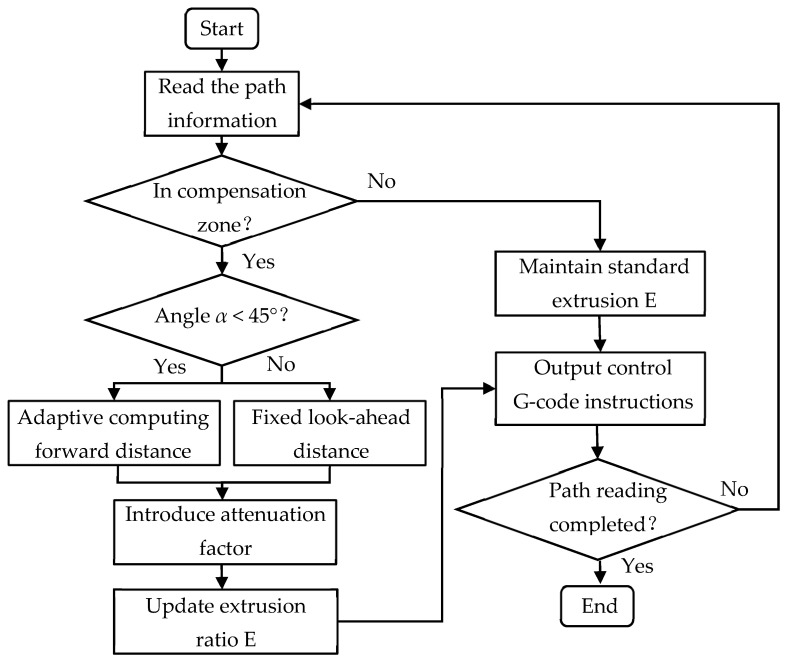
Flowchart of the forward algorithm based on the Sech attenuation factor.

The term $\frac{d}{D_{la}}$ in the equation represents the normalized variable x, ensuring that the attenuation factor always falls within the range of [0, 1]. $M_{max}$ represents the constant maximum flow rate; $M_{min}$ represents the set minimum flow rate, which is set at 0.1 in this design. The control process of M(d) extrusion quantity is shown in Figure 2b. When the nozzle enters the neighborhood with the point identified as a sharp corner as the center, and the look-ahead distance as $D_{la}$, it is determined to enter the gradient control area. The look-ahead control process based on the Sech attenuation factor is shown in Figure 3.

## 3. Control System and Slicing Software

### 3.1. Five-Axis Control System

This project utilized TwinCAT 2.0 (version 2.11.2305, Beckhoff Automation GmbH & Co. KG, Verl, Germany) to build a PC-based five-axis simultaneous-motion additive manufacturing system.

#### 3.1.1. Control System Design

**Figure 4:**
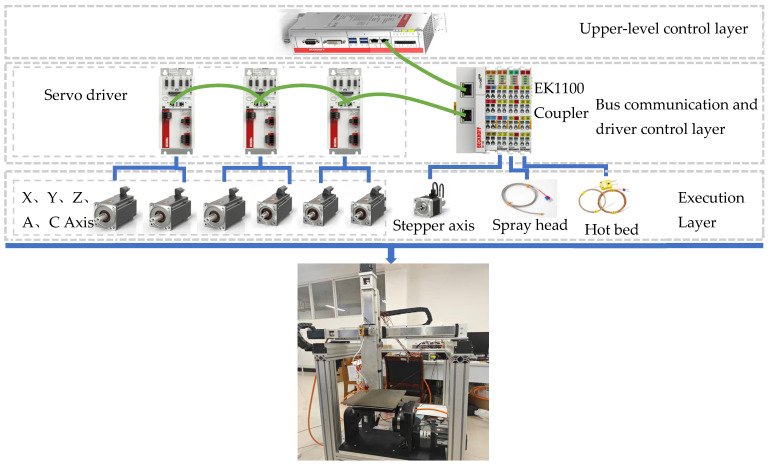
Hardware topology diagram of the five-axis control system based on EtherCAT bus.

The hardware topology structure of the control system is shown in Figure 4. It mainly consists of the upper computer control layer, the bus communication and drive control layer, and the actuator layer. It should be noted that the control hardware used in this system, including the industrial PC, servo drives, coupler, I/O modules, stepper drive module, and motors, was sourced from Beckhoff Automation GmbH & Co. KG, Verl, Germany.

Upper-level control layer: The core controller is selected as the Beckhoff C6920-0060 embedded industrial control computer. This model is equipped with a high-performance processor and runs the TwinCAT CNC real-time kernel. Its main responsibility is to receive the five-axis G-code instruction stream generated by the slicing software, perform the underlying interpolation operation and position closed-loop control through the NC kernel, and be responsible for the PLC logic scheduling of the entire machine.

Bus communication and drive control layer: Three Beckhoff AX5206 dual-channel servo drives and an EK1100 coupler are used, connected through the EtherCAT bus. The AX5000 series drives have high-speed current-loop control capabilities and can precisely execute the periodic position instructions issued by the IPC. Through the EK1100 coupler, the EL7031 stepper drive module, EL3314 thermocouple input module, and EL2008 digital output module are expanded, respectively, to drive the AS1020 extruder stepper motor for smooth filament extrusion control, real-time collection of nozzle and hot bed temperature feedback, and control of solid-state relays through PWM, pulse-width modulation, signals, achieving closed-loop temperature control.

Execution layer: The experimental platform selected the Beckhoff AM8031 and AM8052 series high-performance servo motors as the power sources for the feed axes, and the extrusion axis used the AS1020 stepper motor. Among them, the AM8052 motor is used to drive the Y linear axis with a large load and the A rotation axis; the AM8031 motor, due to its smaller weight, is used to drive the X, Z linear axes with small loads and the C rotation axis, ensuring the dynamic response and positioning accuracy during the printing process. The AS1020 stepper motor is driven by an integrated EL7031 stepper motor terminal module, specifically for controlling the feeding and retraction of the extruder stepper motor.

#### 3.1.2. Five-Axis Kinematic Modeling

**Figure 5:**
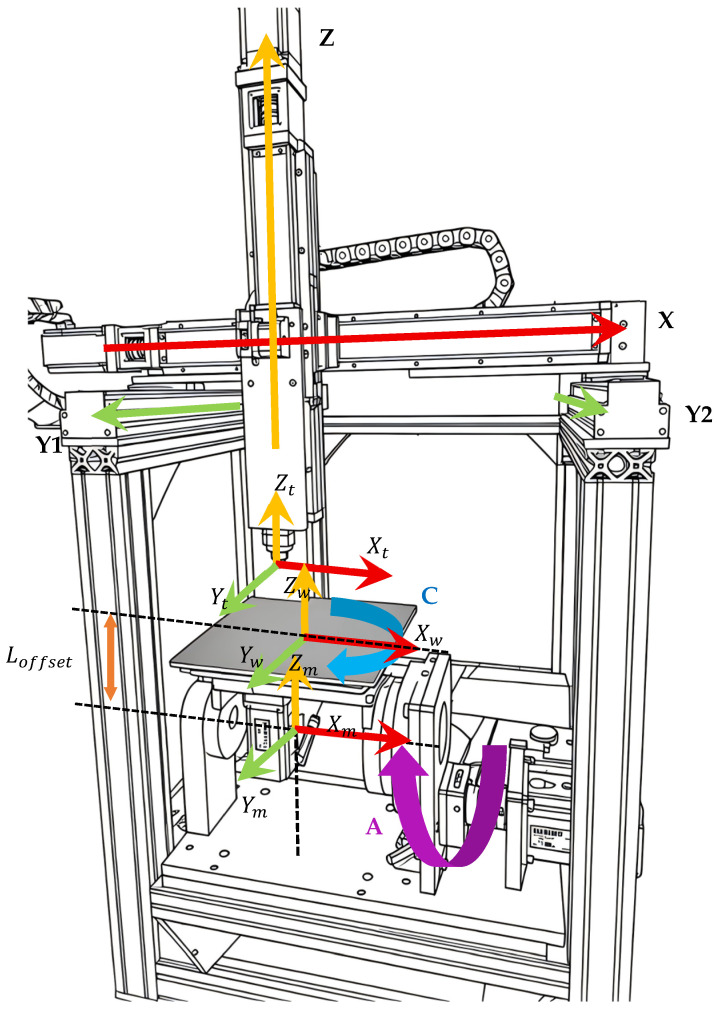
Five-axis coordinate system definition and schematic diagram of rotation axis direction.

This experimental platform adopts a dual-rotary-table AC cradle-type five-axis structure that was independently designed. This structure has the characteristics of good rigidity and fast dynamic response, enabling the printing of complex surfaces without interference. To achieve the accurate mapping of slice data to the motion instructions of each axis, a kinematic model of the machine tool based on the multi-body system theory needs to be established. The topological structure of the machine tool can be regarded as two open-loop motion chains starting from the bed, as shown in Figure 5: The tool motion chain passes through the Y-axis slide saddle, X-axis slide table, Z-axis slide base, and finally reaches the printing nozzle, indicating that the three translational axes are orthogonally superimposed and control the position of the nozzle in space; the workpiece motion chain passes through the A-axis cradle bracket, A-axis cradle, and C-axis turntable and finally reaches the workpiece. Among them, the A-axis rotates around the X-axis, and its positive direction conforms to the right-hand rule, but in the mechanical structure of this equipment, the main working range of the A-axis is negative angles; the C-axis rotates around the Z-axis and is mounted on the A-axis, and the two rotating axes control the posture of the workpiece through a series connection.

To describe the relative positions of each voxel in the motion chain, the system established three core coordinate systems. The machine coordinate system $\left(X_{m},Y_{m},Z_{m},\right)$ serves as the absolute reference benchmark of the system, with its origin set at the geometric intersection point of the rotation center lines of the A-axis and the C-axis; the workpiece coordinate system $\left(X_{w},Y_{w},Z_{w},\right)$ is established at the geometric center of the workpiece’s bottom surface; the vertical offset from the machine coordinate system to the workpiece coordinate system is $L_{offset}={\left[0,0,L\right]}^{T}$. The tool coordinate system $\left(X_{t},Y_{t},Z_{t},\right)$ has its origin at the center of the nozzle tip, and the $Z_{t}$ axis is along the axis of the nozzle.

Based on the above motion chain analysis, the motion relationship between each axis is described using the homogeneous transformation matrix. Let $P_{w}={\left[x_{w},y_{w},z_{w},1\right]}^{T}$ be the homogeneous coordinate of any point on the workpiece in the workpiece coordinate system, which is the original tool position point generated by the slice path planning. Then, the closed-loop motion chain transformation equation from the workpiece coordinate system to the tool coordinate system is:

(5) $$P_{t}={\left(T_{Y}\cdot T_{X}\cdot T_{Z}\right)}^{-1}\cdot T_{A}\cdot T_{C}\cdot (P_{w}+L_{offset})$$

Among them, $T_{A}$ and $T_{C}$ are the rotation transformation matrices for the A-axis and C-axis, respectively, and their specific expressions are as follows:

(6) $$T_{A}=\left[\begin{array}{cccc} 1 & 0 & 0 & 0\\ 0 & cos\theta_{A} & -sin\theta_{A} & 0\\ 0 & sin\theta_{A} & cos\theta_{A} & 0\\ 0 & 0 & 0 & 1 \end{array}\right],T_{C}=\left[\begin{array}{cccc} cos\theta_{C} & -sin\theta_{C} & 0 & 0\\ sin\theta_{C} & cos\theta_{C} & 0 & 0\\ 0 & 0 & 1 & 0\\ 0 & 0 & 0 & 1 \end{array}\right]$$

The translation axis transformation matrix $T_{XYZ}=T_{X}\cdot T_{Y}\cdot T_{Z}$ describes the linear displacement relationship of the tool relative to the origin of the machine tool. Since the X, Y, and Z axes are mutually orthogonal, their combined transformation matrix can be expressed as:

(7) $$T_{XYZ}=\left[\begin{array}{cccc} 1 & 0 & 0 & x\\ 0 & 1 & 0 & y\\ 0 & 0 & 1 & z\\ 0 & 0 & 0 & 1 \end{array}\right]$$

In the equation, x, y, and z represent the real-time displacement amounts of each linear axis relative to the machine’s zero point. This kinematic model constructs an accurate coordinate system transformation relationship by measuring the structural offset vector $L_{offset}$ from the origin of the machine coordinate system to the origin of the workpiece coordinate system. This ensures that the G-code path generated by slicing can be accurately mapped onto the working space of the machine.

#### 3.1.3. PLC Control Program Development

**Figure 6:**
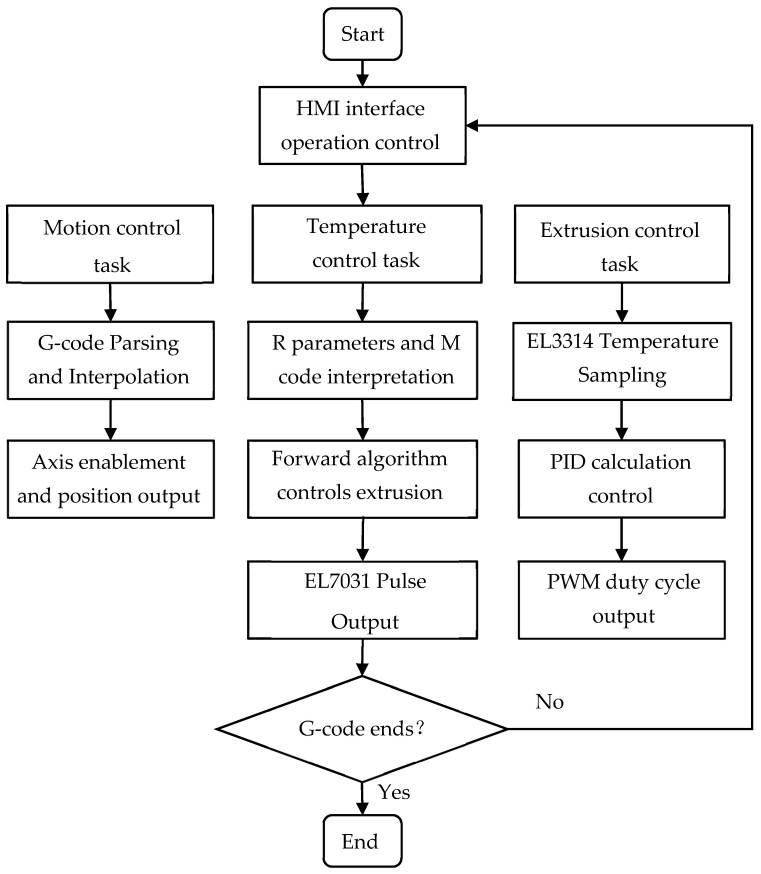
TwinCAT control system operation flow chart.

The software development of the control system mainly relies on the TwinCAT 2.0 development environment, covering NC axis parameter configuration, PLC logic control programs, and human–machine interaction interface (HMI) design. In the System Manager, detailed configurations were made for the X, Y1, Y2, Z, A, and C axes, including setting encoder resolution, electronic gear ratio, maximum speed, and soft limit parameters. Especially for the dual Y-axis structure, a master–slave coupling relationship was established at the NC layer to ensure strict synchronization of the Y1 and Y2 axes during high-speed movement, avoiding mechanical distortion. The PLC program was written in Structured Text (ST) language, and the core logic included system state machine management, the motion control function block call, and look-ahead algorithm control. The operation flow of the control system is shown in Figure 6.

The motion control section utilizes PLCopen standard function blocks provided by TwinCAT NC I (such as MC_Power, MC_MoveAbsolute, and MC_MoveVelocity) for axis activation, homing, and point-to-point positioning. For coordinated five-axis printing tasks, the G-code interpreter parses motion commands via the NC interface and executes multi-axis interpolation. The temperature control logic, as a key part of additive manufacturing, designs an independent PID temperature control function block. This function block reads the nozzle and hot bed temperature values collected by EL3314 in real time, compares them with the set target values, and outputs a PWM signal with an adjustable duty cycle to the EL2008 module to drive the heating elements to work. In addition, for the look-ahead adaptive angle extrusion process, the HLI interface is used to detect the R parameter and the M code trigger signal in the G-code in real time, where the R parameter transmits the look-ahead distance and sharp angle coordinates, and the M code transmits the signals entering and leaving the look-ahead region. When the M code entering the look-ahead area is detected, the function block calculates the Euclidean distance between the nozzle and the sharp corner based on the real-time feedback positions (x, y, z) of each axis at the moment, and then substitutes this value into the hyperbolic cosecant attenuation model to determine the speed modification rate of the extrusion motor.

### 3.2. Development of Five-Axis Slicing Software

For the angle-adaptive look-ahead control algorithm proposed in this study, commercial slicing software cannot directly embed corner feature information into the G-code, nor can it readily accommodate the custom R-parameter interface required by the Beckhoff TwinCAT system. Therefore, this design independently developed a slicing path planning software that is compatible with the five-axis additive manufacturing platform. It is particularly important to note that the extrusion accumulation prediction model established in this study mainly focuses on the geometric features within the XY plane. Under the current algorithm framework, the model assumes that the printing path has a constant Z-axis height within the same layer, that is, it does not consider the non-uniform thickness slicing or the flow fluctuations caused by the drastic changes in the Z-axis during curved surface printing. Based on this physical assumption, in the algorithm verification stage, this study mainly enabled the software’s three-axis printing mode for experiments to achieve the precise verification of the look-ahead compensation effect within the XY plane. As for the coupling influence of the XYZ three-axis linkage on the accumulation phenomenon, it will be the key research direction of this topic in the future.

#### 3.2.1. Slicing Software Development Process

**Figure 7:**
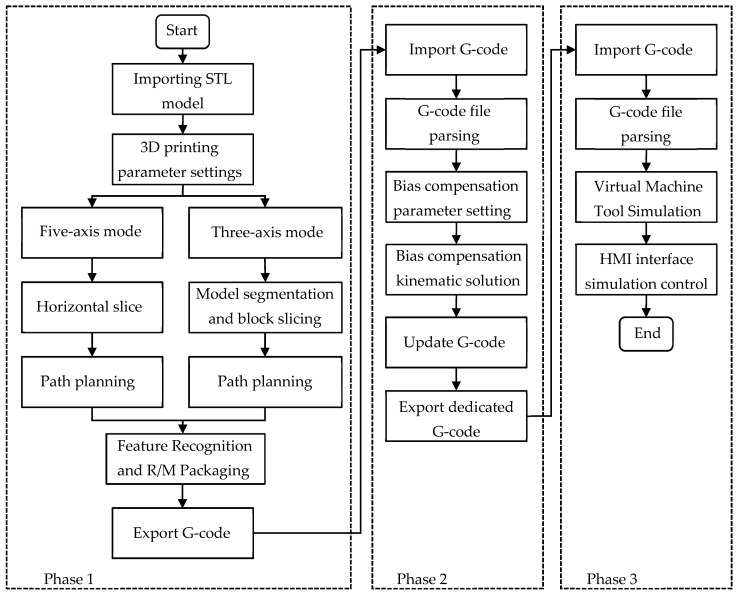
Five-axis slicing software processing flowchart.

The slicing software was developed following the workflow of geometric segmentation, initial kinematic solving, offset-compensated kinematic post-processing, and simulation verification. During the operation, it is mainly divided into three functional stages, and the overall process is shown in Figure 7.

Phase 1: Model segmentation and slice setup. This phase mainly involves geometric path planning and look-ahead feature extraction. After the user imports the STL model and configures the process parameters, the system performs adaptive slicing and path planning. During this process, the look-ahead algorithm module is activated, which identifies the sharp corner features in the path, calculates the corresponding state parameters, and encapsulates them as M code and R parameters. Finally, the system outputs an initial G-code file based on the workpiece coordinate system. This file contains complete geometric paths and process feature information, but does not yet include kinematic offsets specific to the machine tool structure.

Phase 2: Post-processing of 5-axis G-code. During the kinematic calculation in the first phase, the origin of the machine tool coordinate system was set at the geometric intersection point of the rotation centers of the A-axis and C-axis ($(X_{m},Y_{m},Z_{m}$) in Figure 5); however, in practical engineering applications, due to mechanical assembly errors, differences in the thickness of the printing platform, or different specifications of cradles, there is often a vertical offset between the center of the workpiece bottom surface (($X_{w},Y_{w},Z_{w}$) in Figure 5) and the ideal rotation center ($L_{offset}$ in Figure 5). This software allows users to adjust the compensation amount arbitrarily according to the actual mechanical structure through an open configuration interface for the offset vector in the post-processing algorithm, enabling the same set of post-processing logic to adaptively generate precise motion instructions suitable for different machine tool hardware, significantly enhancing the universality of the system. During this process, the forward-looking feature parameters (R/M code) are directly retained due to their geometric invariance. Finally, in the post-processing stage, a dedicated NC program adapted to the specific machine tool structure, including offset compensation and complete feature information, will be generated.

Phase 3: Printing simulation and path preview. To ensure the safety and feasibility of the generated G-code, the software provides a three-dimensional simulation environment. The system loads the kinematic model of the machine tool, parses the generated G-code, and simulates the actual movement trajectory of the print head within the machine tool’s working space. Users can visually check for any collision interference, axis overtravel, or unexpected violent movements.

#### 3.2.2. Five-Axis Kinematics

The core of the coordinate transformation for five-axis simultaneous-motion printing lies in converting the slice path points $\left(x_{w},y_{w},z_{w}\right)$ in the workpiece coordinate system into the motion instructions for each axis of the machine tool $\left(X,Y,Z,A,C\right)$. This software adopts a post-processing algorithm based on an inverse kinematics solution.

During the process of obtaining the inverse solution of the rotational joint angles, in order to ensure that the nozzle axis remains perpendicular to the slicing layer throughout the printing process, it is necessary to align the normal vector $\underset{n_{w}}{\to }={\left[n_{xw},n_{yw},n_{zw}\right]}^{T}$ of the slicing plane generated by the slicing path planning in the workpiece coordinate system (WCS) with the Z-axis direction ${\left[0,0,1\right]}^{T}$ of the machine coordinate system through the rotation of the A-axis and the C-axis. According to the rotation matrix $T_{A}$ and $T_{C}$ defined by Equation (6), the attitude transformation equation can be expressed as:

(8) $$T_{A}(A)\cdot T_{C}(C)\cdot \vec{n_{w}}={\left[0,0,1\right]}^{T}$$

Since the A-axis of the experimental machine tool is defined as rotating around the X-axis, the movement of the A-axis can only change the projection direction of the vector in the YZ plane, but cannot eliminate the X-axis component. Therefore, the inverse solution process must first rotate the slicing normal vector $\underset{n_{w}}{\to }$ to the YZ plane through the C-axis and then rotate it to align with the Z-axis through the A-axis. Based on this geometric constraint, the inverse solution equation for the joint angle is as follows:

(9) $$\left\{\begin{array}{c} A=-\arccos(n_{zw})\\ C=90{}^{\circ}-\arctan2(n_{\mathrm{yw}},n_{\mathrm{xw}}) \end{array}\right.$$

After determining the rotation angle (A, C), the compensation motion of the linear axis (X, Y, Z) must be calculated to counteract the displacement caused by the rotation. The compensation motion of the linear axis is calculated based on the closed-loop equation defined in Section 3.1.2. The path point $P_{w}={\left[x_{w},y_{w},z_{w}\right]}^{T}$ in the workpiece coordinate system first adds the structural offset vector $L_{\mathrm{offset}}={\left[0,0,L\right]}^{T}$, and then undergoes rotation transformations for the C-axis and A-axis successively. The motion equation for the movement to the new position $P_{m}={\left[X,Y,Z\right]}^{T}$ in the machine coordinate system is as follows:

(10) $$P_{m}=\left[\begin{array}{c} X\\ Y\\ Z \end{array}\right]=T_{A}(A)\cdot T_{C}(C)\cdot (P_{w}+L_{offset})$$

The workpiece point first undergoes a translation along the structural offset vector $L_{offset}$ to the reference system of the rotation center, and then the rotation transformations along the C-axis and A-axis are carried out successively. By substituting the rotation matrices $T_{A}$ and $T_{C}$ into the above equation and expanding it, the actual execution instructions for the linear axes of the machine tool can be obtained:

(11) $$\left\{\begin{array}{c} X\\ Y\\ Z \end{array}\begin{array}{c} =\\ =\\ = \end{array}\begin{array}{c} x_{w}\mathrm{cosC}-y_{w}\mathrm{sinC}\\ (x_{w}\mathrm{sinC}+y_{w}\mathrm{cosC})\mathrm{cosA}-(z_{w}+L)\mathrm{sinA}\\ (x_{w}\mathrm{sinC}+y_{w}\mathrm{cosC})\mathrm{sinA}+(z_{w}+L)\mathrm{cosA} \end{array}\right.$$

### 3.3. Algorithm Implementation

#### 3.3.1. Sharp Corner Detection

The automatic feature recognition of discrete paths is the core prerequisite for achieving angle-adaptive forward planning. This algorithm performs vectorization processing on the discrete point set generated by STL slicing, simultaneously generating geometric paths and real-time identifying local features. It directly encapsulates the key parameters into the G-code instruction stream as the data source for real-time control by the PLC. The steps are as follows.

1. Vectorized angle extraction

**Figure 8:**
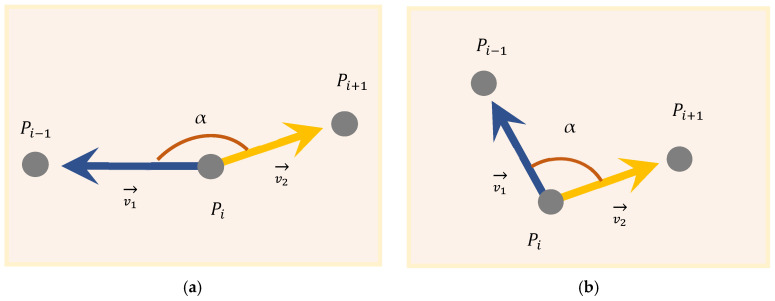
Discrete point vector calculation diagram: (**a**) If angle α is greater than angle $\alpha_{\mathrm{th}}$, no compensation is required for this point; (**b**) if angle α is less than angle $\alpha_{\mathrm{th}}$, compensation is necessary for this point.

The algorithm traverses the discrete point set generated by STL slicing. For any sequence of adjacent points $P_{i-1}$, $P_{i}$, $P_{i+1}$, with the middle vertex $P_{i}$ as the origin, two reverse vectors are constructed: $\vec{v_{1}}=P_{i}\vec{P_{i-1}}$ and $\vec{v_{2}}=P_{i}\vec{P_{i+1}}$, as shown in Figure 8. The interior angle α at this point is calculated in real time using the vector dot product equation:

(12) $$\cos\alpha =\frac{\underset{v_{1}}{\to }\cdot \underset{v_{2}}{\to }}{\left|\underset{v_{1}}{\to }\right|\left|\underset{v_{2}}{\to }\right|},\;\alpha =arccos(clip(cos\alpha ,-1,1))$$

By comparing the calculated internal angle α with the defined compensation angle threshold $\alpha_{\mathrm{th}}$, it is possible to determine whether $P_{i}$ is a point that requires compensation.

2. Sharp Angle Detection

In order to automatically identify the geometric singular points in the path that have the risk of over-extrusion, this algorithm defines $\alpha_{\mathrm{th}}$ determination function S(α) based on the angle threshold a. This operator makes real-time judgments on the local path angle α:

(13) $$S(\alpha )=\left\{\begin{array}{c} 1,\;\alpha <\alpha_{\mathrm{th}}\\ 0,\;\alpha \geq \alpha_{\mathrm{th}} \end{array}\right.$$

In this study, the $\alpha_{\mathrm{th}}$ threshold was set at 160°. The determination method is as follows.

When S(α) = 1, the path is classified as a “sharp corner point”, as shown in Figure 8b. The algorithm will activate the subsequent angle-adaptive look-ahead and Sech gradient control logic.

When S(α) = 0, the path is identified as a smooth, continuous curvature path, as shown in Figure 8a. The geometric overlap between adjacent segments is minimal, and the algorithm maintains constant extrusion to ensure surface consistency.

3. Feature parameter G-code encapsulation

Once the valid sharp corner feature is identified, the slicing software immediately serializes the physical attribute sequence of that point into a set of R parameters and inserts them into the G-code instruction stream through custom M code. The encapsulated data structure is as follows.

R1($D_{la}$): Forward compensation distance indicates how far from the corner point the PLC should start applying speed modulation.

R2($\alpha_{i}$): The geometric internal angle of this point is determined by the compensation method that is enabled.

R3, R4, R5($X_{c},Y_{c},Z_{c}$): The absolute physical coordinates of the sharp corner vertex. This algorithm only considers the rotational extrusion material accumulation phenomenon within the XY plane. The actual parameters used are X and Y values.

#### 3.3.2. Analysis and Control of PLC Feature Parameters

In order to achieve the real-time transmission of look-ahead feature parameters, the system established a data channel from the G-code interpreter to the PLC based on the HLI interface of TwinCAT CNC. When the interpreter reads an instruction line such as N100 M100 R1 = 5.2 R2 = 90 R3 = 100.5 R4 = 200.0 R5 = 0.2, the kernel automatically writes the M code number into the ST_CncSystem structure and maps the R parameters to the R_Param_Float array. The PLC program monitors the M function trigger status by periodically calling the HLI_CncGetMKey function block. Once the corresponding M signal is detected, the PLC immediately extracts the look-ahead distance R1($D_{la}$), the corner angle R2($\alpha_{i}$), and the physical coordinates of the sharp corner R3, R4, R5($X_{c},Y_{c},Z_{c}$) from the interface memory and stores them in the internal queue.

Considering that the pre-reading mechanism of the CNC interpreter causes the execution of M-code to occur earlier than the physical movement reaches its destination, the PLC introduces a First-In-First-Out (FIFO) queue to cache the pre-read feature parameter groups. During each control cycle, the PLC reads the real-time feedback position $P_{act}=(X_{act},Y_{act},Z_{act})$ of each servo axis. Given that this study mainly focuses on the extrusion accumulation phenomenon caused by path turns within the XY plane, and the experimental verification scenario is plane layer-by-layer printing, therefore, when calculating the real-time distance d, the algorithm only considers the projection components of the XY plane:

(14) $$d=\sqrt{{\left(X_{act}-X_{c}\right)}^{2}+{\left(Y_{act}-Y_{c}\right)}^{2}}$$

Based on the current distance d and the characteristic parameter α, the PLC can calculate the speed ratio M(d) of the extrusion motor in real time using Equations (3) and (4). The calculated ratio value M(d) is then immediately applied to the extrusion shaft through the MC_SetOverride function block, dynamically reducing its feed speed. As the nozzle gradually approaches and moves away from the sharp corner, the distance d first decreases and then increases, and the ratio subsequently shows a changing pattern of first decreasing and then increasing, thereby effectively suppressing the over-extrusion and accumulation phenomenon caused by the lag in acceleration and deceleration at the sharp corner. When d is again greater than $D_{la}$, the state machine resets and the extrusion speed returns to 1.

## 4. Experiment

### 4.1. Experimental Equipment

To verify the angle-adaptive forward compensation strategy based on the Sech attenuation curve, our research group has built an open five-axis additive manufacturing experimental platform based on a PC architecture. This platform uses a high-performance industrial control computer as the core controller, and through a high-speed real-time Ethernet bus, it tightly connects the complex path instructions generated by the upper-level slicing software with the underlying servo drive system, providing a hardware foundation for the high-quality formation of complex surface features.

**Figure 9:**
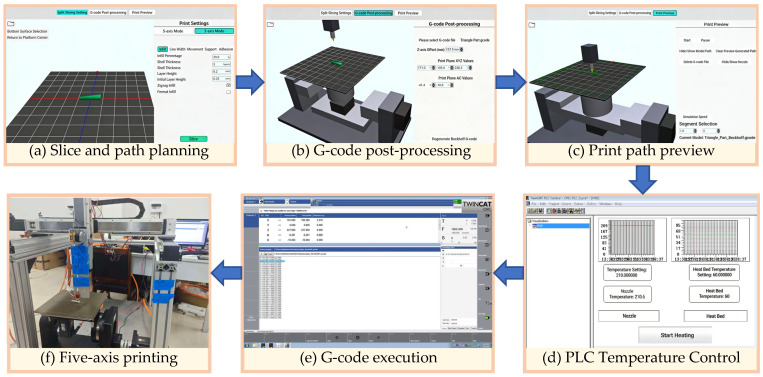
Algorithm verification of printing process diagram.

The control of the entire printing process is shown in Figure 9. First, the five-axis slicing software developed in this paper is run on the PC to plan the path and serialize the parameters for the STL model, as shown in Figure 9a, generating a G-code file that includes geometric paths and R parameters. Offset compensation is first applied to the A/C-axis rotary-center, followed by path simulation to verify the absence of deviation and collision, as shown in Figure 9b,c. The G-code can then be imported into the TwinCAT system of the controller through the file transfer method, and imported into the TwinCAT human–machine application program, as shown in Figure 9d. The nozzle and hot bed temperature are controlled through the PLC human–machine interface, as shown in Figure 9e. After confirming that the nozzle and hot bed have been heated, printing begins in the TwinCAT human–machine application program interface. Figure 9f shows the actual printing process. The NC core layer is responsible for interpreting the G-code and multi-axis interpolation operations, while the PLC logic layer reads the forward parameters in real time, uses the Sech function to calculate the current extrusion speed modulation coefficient, and dynamically corrects the feed rate of the extruder, thereby achieving a complete closed-loop verification from the slicing algorithm to the physical manufacturing at the hardware level.

### 4.2. Manufacturing Parameter Settings

In this experiment, PLA filament from Creativity (Shenzhen, Guangdong, China) was used as the printing material. To verify the performance of the forward algorithm under normal printing conditions, the slicing parameters were set to the typical medium-speed printing mode. The specific parameter configuration is shown in Table 1.

The parameters of the look-ahead algorithm were set based on the theoretical derivation results in Section 2, have been preliminarily tested, and are now ready for application. The parameters can be further fine-tuned according to the actual hardware system. The specific algorithm parameters are shown in Table 2.

### 4.3. Printing Experiments and Analysis

In order to comprehensively verify the effectiveness of the proposed angle-adaptive forward compensation algorithm in different geometric features, a triangular feature test sample with extreme angle variations was designed. This sample includes three typical corner features, aiming to simulate various path conditions that may be encountered in the actual printing of complex curved surfaces, in order to test the algorithm’s optimization effect on the material accumulation at corners.

**Figure 10:**
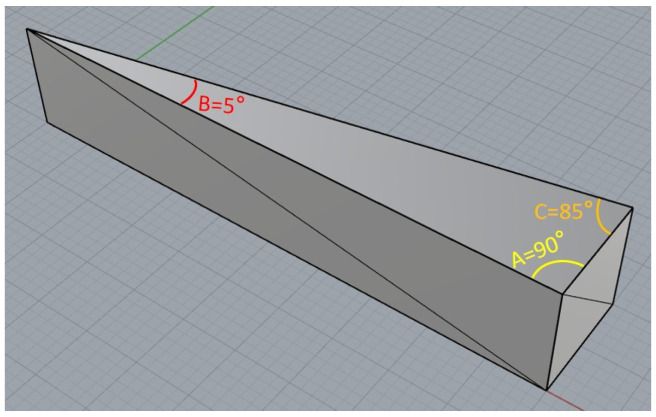
Compensation algorithm test sample.

As shown in Figure 10, the verification sample we designed is a thin-walled structure of a non-equilateral triangle. The geometric characteristics of its three vertices are as follows:

- Vertex A forms a 90° right angle simulating the most common regular geometric feature. At this angle, the direction of the path undergoes a significant change, but it does not reach an extremely sharp degree, which is used to verify the smooth transition stability of the algorithm under standard conditions.
- Vertex B is a 5° extremely acute angle simulating the extreme sharpness of thin-walled features or blade-like edges. Here, the printing nozzle needs to perform an almost 180° reverse return movement, and the two paths are extremely close, making it prone to severe “over-extrusion” or “collapse” due to heat accumulation and excessive extrusion. This is a key point for testing the ultimate performance of the forward-looking algorithm.
- Vertex C is an acute angle of 85°: It lies between a right angle and an extremely acute angle, simulating the characteristics of a typical acute angle (greater than 45°), and is used to verify the generality and robustness of the algorithm within the intermediate angle range.

In the experiment, two sets of control strategies were respectively adopted for comparative printing:

- Experimental Group ①: Applied the angle-adaptive forward compensation algorithm proposed in this paper, which can adjust the speed in real time according to the path angle;
- Control Group ②: Utilized the traditional print speed control strategy without any dynamic flow compensation.

**Figure 11:**
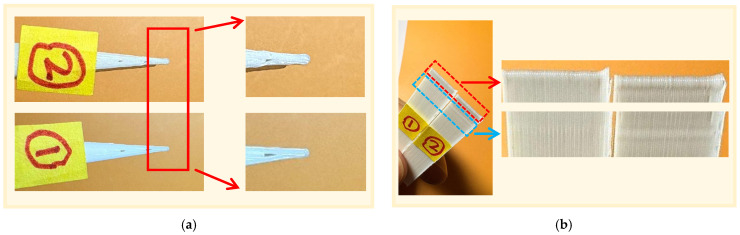
Limit acute angle 5° observation result diagram: (**a**) Horizontal view observation result diagram; (**b**) side view observation result diagram.

Figure 11 presents the microscopic observation results at the most challenging 5° extremely acute angle feature. On the left side of the Figure, label ① represents the experimental sample with the forward-looking algorithm enabled, while label ② on the right represents the control sample without the algorithm enabled. First, observe the top view in Figure 11a. It can be clearly seen that the control group ② shows a distinct dome-shaped bulge at the tip of the sharp angle. This is because the nozzle decelerates sharply on the extremely short return path, and the extruder fails to respond promptly to the change in flow rate, causing a large amount of molten material to accumulate at the tip due to inertia, severely damaging the geometric profile of the sharp angle. In contrast, the tip of the sharp angle of experimental group ① has clear and sharp lines, with no excess material overflowing, and the corner transition is smooth and well-defined.

In the side view of Figure 11b, the side wall of control group ② shows a significantly raised layer pattern. Due to the high degree of path overlap at the sharp corners, the residual heat and material compression within the inner circle directly affect the outer circle, resulting in a clear accumulation effect of the second circle pattern on the surface, forming a series of raised edge lines. However, experimental group ① basically eliminated this phenomenon. The layer pattern on the side wall is smooth and uniform, with a high degree of integration between layers. Moreover, because the algorithm precisely and dynamically regulates the flow rate, the printing process of the second circle pattern does not have an effect on the surface to cause raised protrusions. The overall surface presents a high-quality smoothness.

**Figure 12:**
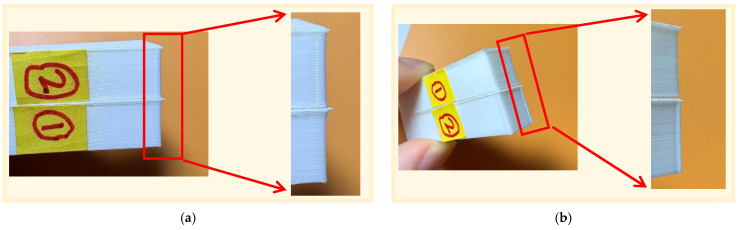
Right angle 90°and acute angle 85° observation result diagram: (**a**) Observation result diagram of acute angle 85°; and (**b**) observation result diagram of right angle 90°.

For the relatively common geometric features of an 85° acute angle and a 90° right angle, the experimental results also verified the superiority of the algorithm. As shown in Figure 12, the side view comparison effect under these two angles is presented. In Figure 12, the control group sample marked as ② still has periodically raised layer patterns at the corner, indicating that even at non-extreme angles, the inertia lag of the mechanical system still has a negative impact on the formation quality. Meanwhile, the experimental group sample marked as ① demonstrated excellent contour control ability when handling these two corner transitions. Whether it is an 85° or 90° corner transition, it maintained a high degree of smoothness, effectively eliminating the raised layer patterns, proving that this algorithm can effectively suppress the material accumulation and significantly improve the forming accuracy within different angle ranges.

**Figure 13:**
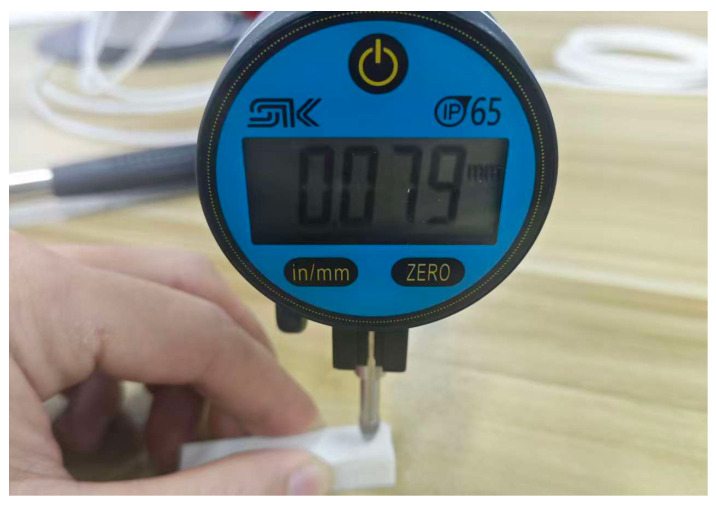
Electronic digital micrometer measurement diagram.

Although the above microscopic observation results directly demonstrated the effectiveness of the compensation algorithm in suppressing material accumulation, in order to overcome the limitations of purely visual qualitative assessment, this study further introduced a high-precision digital dial indicator (SYNTEK, model 59BFF12SK, Huzhou, China) with a resolution of 0.001 mm to measure the surface protrusions. Strict quantitative measurements were further performed on the local geometric protrusion increments at the three representative corner angles (5°, 85°, and 90°), as shown in Figure 13.

During the measurement operation, first, the measuring tip of the micrometer is placed at a position near the corner of the sample where there is no path overlap. The actual height at this point is used as the reference zero point for calibration and zeroing. Subsequently, the measuring tip is slowly moved to the geometric vertex with the sharpest corner of the corner. Considering the possible slight elastic indentation of the measuring tip’s preload force on the polymer material during contact measurement, the reading process maintains a consistent contact state to record stable peak data. The difference between this peak and the reference zero point is defined as the protrusion increment caused by over-extrusion at the corner. For each angle feature (5°, 85°, and 90°) in the experimental group ① and the control group ②, the above baseline calibration and peak reading process were repeated at three different positions at the same layer height to eliminate accidental errors and calculate reliable average increments. The measurement results are summarized in Table 3.

As shown in Table 3, in the un-compensated control group ②, the average protrusion increments for 5°, 85°, and 90° turning angles reached 0.073 mm, 0.061 mm, and 0.064 mm, respectively. In this experiment, the layer thickness was set at 0.2 mm, and these un-compensated local over-extrusion protrusions had already exceeded 30% of the layer thickness. In the layer-by-layer stacking additive manufacturing process, as the number of printing layers increases, this error positively accumulates rapidly and inevitably leads to rigid scraping and interference between the extrusion nozzle and the formed structure. After the introduction of the Sech-curve-based adaptive look-ahead compensation, the average bulge increase in the three angles in the experimental group ① was strictly suppressed between 0.003 mm and 0.005 mm, accounting for only about 2% of the layer thickness, and the reduction rate of protrusion at each angle exceeded 90%. This quantitative data spanning from extremely acute angles to right angles further confirms that this algorithm has a high degree of geometric feature adaptability, can effectively solve the coupling interference caused by overlapping paths and sudden speed changes, and thus ensures the dimensional consistency of the extreme structure printing process.

## 5. Conclusions

Aiming at the problem of material over-accumulation and contour errors at the corner caused by path curvature mutations in the process of fused deposition forming, an angle-adaptive look-ahead compensation algorithm based on the Sech curve attenuation is proposed and verified. A Beckhoff five-axis 3D-printing experimental platform was built, and a slicing path planning software was developed to generate dedicated G-code. Feature samples, including extreme acute angles, ordinary acute angles, and right angles, were designed for comparative experiments to solve the problem of material accumulation at the corners. The experimental results show that this algorithm has good universality and robustness, and it can achieve smooth and continuous motion trajectory planning under different geometric features, significantly reducing material accumulation and contour errors at corners. The main contributions of this chapter are as follows:

(1) An angle-adaptive compensation strategy based on the Sech curve attenuation was proposed and verified. For complex features such as extremely acute angles, a mapping model between the path angle and the adaptive look-ahead distance was established, enabling smooth decoupling control of the corner speed and extrusion flow rate.
(2) A 3D-printing five-axis control system based on Beckhoff TwinCAT architecture was constructed. The look-ahead algorithm was calculated at the PLC layer and synchronized interpolation was performed at the NC layer using the TwinCAT 2 real-time kernel.
(3) An integrated software package for five-axis slicing and path planning was developed. A slicing software that integrates model processing, path planning, G-code generation, and post-processing has been independently developed. This software solves the problem that traditional software cannot generate dedicated five-axis G-code containing R/M extended parameters. At the same time, the post-processing module of the software integrates a dynamic offset correction function. Users can configure it in real time according to the structural parameters, such as the turntable center offset of different five-axis equipment, so that the NC code generated by this software can quickly adapt to different specifications of five-axis linkage manufacturing platforms.

Although the angle-adaptive look-ahead algorithm proposed in this paper has achieved remarkable results in resolving path overlap and excessive corner accumulation within the XY plane, it still has certain limitations and needs to be further improved in subsequent research. Currently, the algorithm mainly focuses on feature compensation for planar paths and has not fully considered the impact of Z-axis direction changes on material extrusion. In actual five-axis simultaneous-motion printing, for parts with spatially variable curvature characteristics such as bent pipes, due to the variation in slice thickness along the normal surface direction, only planar compensation has difficulty with completely eliminating Z-direction material accumulation or under-extrusion defects. Therefore, in the future, further research on flow compensation algorithms in spatial dimensions can be conducted, introducing adaptive layer thickness adjustment and Z-axis motion compensation mechanisms to achieve more comprehensive control of the formation quality of complex spatial paths.

## Figures and Tables

**Table 1:** Slice parameter configuration table.

Parameter Category	Parameter Name	Set Value/Description	Organization
Basic process	Nozzle diameter	0.4	mm
	Layer height	0.2	mm
	Line width	0.42	mm
	Printing speed	30	mm/s
Temperature	Spray head temperature	210	°C
	Heating bed temperature	60	°C

**Table 2:** Look-ahead algorithm parameter configuration table.

Symbol	Parameter Name	Set Value/Description	Organization
$D_{la}$	Look-ahead compensation distance	Adaptive computing	mm
$\alpha_{th}$	Sharp corner detection threshold	160	Degree
$M_{min}$	Minimum extrusion ratio	0.1	—
k	Sech shape factor	4.0	—

**Table 3:** Measurement results of protrusions at different turning points.

Corner Angle	Group	Position 1	Position 2	Position 3	Average Bulging
5	Experimental Group ①	0.006	0.004	0.003	0.004
	Control Group ②	0.079	0.07	0.071	0.073
85	Experimental Group ①	0.005	0.006	0.004	0.005
	Control Group ②	0.056	0.067	0.061	0.061
90	Experimental Group ①	0.001	0.003	0.004	0.003
	Control Group ②	0.068	0.07	0.055	0.064

## Data Availability

The data presented in this study are available on request from the corresponding author. The data are not publicly available due to the need to protect the rights and interests of the authors and avoid plagiarism.
